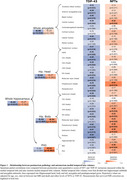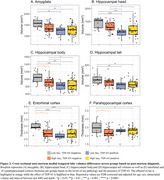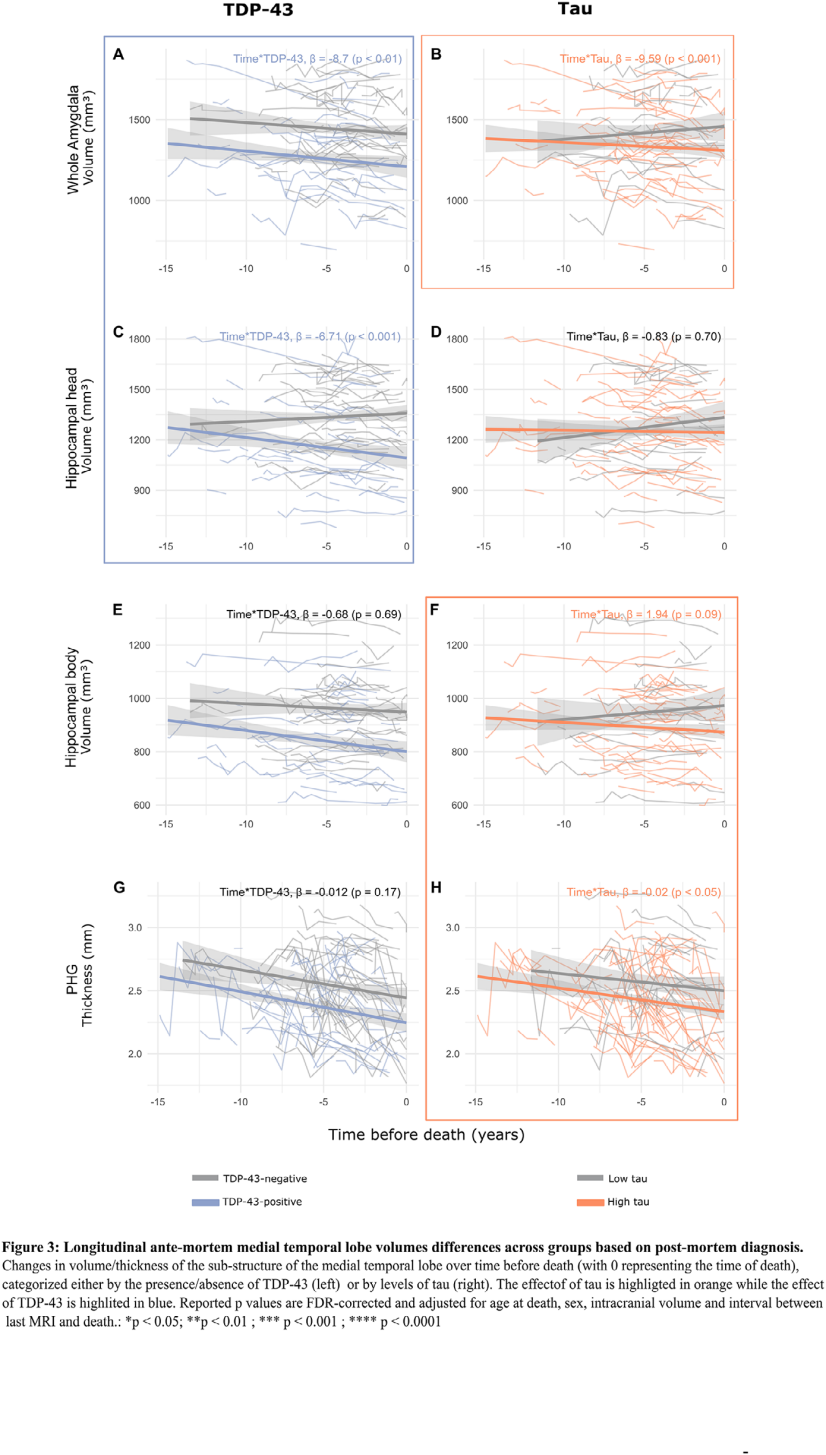# Specific atrophy patterns distinguish tau and TDP‐43 pathology: a longitudinal MRI ante‐mortem study

**DOI:** 10.1002/alz70862_110863

**Published:** 2025-12-23

**Authors:** Yasmine Salman, Julia Goloubeva, Lara Huyghe, Lisa Quenon, Sandra O. Tomé, Bernard J Hanseeuw

**Affiliations:** ^1^ Institute of Neuroscience, UCLouvain, Brussels Belgium; ^2^ UCLouvain, Brussels, Brussels Belgium; ^3^ Leuven Brain Institute, Leuven Belgium; ^4^ Laboratory of Neuropathology, KU Leuven, Leuven, Vlaams‐Brabant Belgium; ^5^ Massachusetts General Hospital, Gordon Center for Medical Imaging and the Athinoula A. Martinos Center for Biomedical Imaging, Boston, MA USA

## Abstract

**Background:**

Alzheimer’s disease (AD) cases often present with TDP‐43 inclusions at autopsy, suggesting comorbid Limbic‐predominant Age‐related TDP‐43 Encephalopathy (LATE). These patients show smaller hippocampal volume and more severe cognitive decline than ‘pure’ AD patients. Distinguishing the contributions of both pathologies to neurodegeneration is crucial for treatment development; but is challenging as both pathologies affect the medial temporal lobe (MTL), share similar clinical symptoms and there is no in‐vivo biomarker for TDP‐43. We aimed to disentangle the relative contribution of tau and TDP‐43 pathologies to the atrophy of MTL substructures in AD patients.

**Methods:**

We conduced antemortem cross‐sectional and longitudinal MRI analyses in participants with neuropathological data obtained at post‐mortem. Participants were selected from the ADNI database (*N* = 85) and grouped according to Braak stages (Low tau [0‐III] or High Tau [IV‐VI]) and the presence of TDP‐43 in the MTL. Statistical analyses included cross‐sectional correlations, group analyses, and liner‐mixed models predicting volume changes before death. All models were adjusted for MRI‐death‐interval, age, sex and intracranial‐volume.

**Results:**

TDP‐43 was mostly associated with the volume of the hippocampal head (*R* = ‐0.47; *P* < 0.01, Figure 1) while NFTs were associated with the thickness of the parahippocampal gryrus (PHG; *R* = ‐0.41; *P* < 0.01, Figure 1). Consistently, among individuals with low levels of tau, TDP‐43‐positivity was associated with atrophy in all MTL structures except the parahippocampal cortex (PHC, Figure 2). In contrast, in TDP‐43‐negative individuals, high tau was associated with atrophy in the hippocampal body (*β* = ‐137mm^3^, *P* < 0.05), entorhinal cortex (*β* = ‐0.49mm, *P* < 0.01) and PHC (*β* = ‐0.28mm, *P* < 0.01, Figure 2). Longitudinal analysis showed that the hippocampal head volume reduction over time was faster in the presence of TDP‐43 in the MTL (*β* = ‐6.71mm^3^/year, *P* < 0.001, Figure 3) while the PHG thickness reduction over time was faster with higher Braak stages (*β* = ‐0.02mm/year, *P* < 0.05, Figure 3).

**Conclusion:**

We observed an anterior‐posterior effect of TDP‐43 and tau on the MTL with TDP‐43 affecting anterior MTL volumes and tau posterior MTL volumes. Hippocampal head atrophy could help distinguish between AD patients with/without comorbid LATE.